# Data on the students’ body management and ego-identity status at Kermanshah University of Medical Sciences, Iran

**DOI:** 10.1016/j.dib.2018.10.024

**Published:** 2018-10-09

**Authors:** Yahya Safari, Nasrin Yoosefpour

**Affiliations:** Research Center for Environmental Determinants of Health (RCEDH), Kermanshah University of Medical Sciences, Kermanshah, Iran

**Keywords:** Body management, Ego-identity status, University students, KUMS

## Abstract

Physical attractiveness is one of the topics requiring new standards in the modern age, whereby the relationships between individuals are formed. The present data article aimed to evaluate the students’ body management and ego-identity status at Kermanshah University of Medical Sciences (KUMS), Iran in 2017. The statistical population consisted of 4200 students at KUMS. To collection of this dataset, 360 students were selected using the Krejcie and Morgan Sampling Formula, and the sampling method was cluster sampling. As for data collection, the Ego-Identity Status (EOM-EIS) Questionnaire and Multidimensional Body–Self Relations Questionnaire (MBSRQ) were utilized. The obtained data indicated that KUMS students enjoyed proper body management.

**Specifications table**

**Table:** 

Subject area	Social sciences
More specific subject area	Health sciences
Type of data	Tables
How data was acquired	The statistical population of the study consisted of all students at KUMS (N=4200), of whom 360 persons were selected as the sample population. As for data collection, the Ego-Identity Status (EOM-EIS) Questionnaire (Benion and Adamz, 1989) and Multidimensional Body–Self Relations Questionnaire (MBSRQ) (Cash et al., 2015) were utilized [1], [2].
Data format	Raw, analyzed
Experimental factors	All responses were scored based on Likert scale, and unanswered questions or invalid responses in questionnaires were regarded as missing data.
Experimental features	The relationship between body management and identity base was evaluated using the linear regression test.
Data source location	Kermanshah, Iran
Data accessibility	Data are included in this article
Related research article	A. Kumru, R.A. Thompson, Ego identity status and self-monitoring behavior in adolescents, J. Adolesc. Res. 18(2003) 481–95. Published [3]

**Value of the data**

• The data of the present data article can provide the faculty members and university staff with knowledge about the status of their body management, evaluation of students’ identities, proper and deft planning to prevent problems such as identity-related crises in students, recognition of proper ways for improving the quality of students’ identities. Therefore, educational and clinical services can provide such opportunities.
• Psychotherapy can benefit from the ego-identity approach more than any other psychological domain. Moreover, given, the formation of the identity of authorities and the current situation in this field, paying attention to ego-identity approach is very useful for treatment, and its neglect can bring about harmful consequences.
• Body culture refers to the increasing importance of the fact that body is a focal point for interactions and self-reflection of one׳s own. Therefore, body management should be evaluated as a fundamental issue. So, the data article aimed to evaluate the correlation between students’ body management and ego-identity status at Kermanshah University of Medical Sciences (KUMS), Iran in 2017.
• So far, no such data collection has been conducted on the subject under discussion in KUMS. Therefore, the data from the present study can be useful for KUMS students and serve as the basis for future studies in term of methodology and obtained raw data of it.

## 1 Data

In this study, the comments of 266 students about body management and its relationship with ego-identity were evaluated. The average age of participants in the study was 22.57 with a standard deviation of 3.58. The age range varied from 18–33. Moreover, women accounted for the highest percentage of samples (56%). Further, single subjects made up just over three quarters of the whole samples (80.8%). Not to mention, around 95.5% of them earned less than $1000 per month. In addition, 91.7% of the samples were non-medical students (Table 1). As can be seen in Table 2, the mean scores of ego-identity and body management measured 187.61 and 153.12, respectively. The mean of ego-identity status, body management and its subscales by gender variable was showed in Table 3.

**Table 1:** Frequency distribution of demographic variables in research units.

**Variable**		**Frequency**	
		**Number**	**Percent**
Gender	Male	117	44
	Female	149	56
Marital status	Single	215	80.8
	Married	51	19.2
Income (Tooman)	Less than 3 million	254	95.5
	More than 3 million	12	4.5
Job	Student of Medical Sciences	22	8.3
	Other students	244	91.7

**Table 2:** The means and standard deviations of ego-identity, body management and their dimensions.

**Variables**	**Mean**	**SD**	**Min**	**Max**
**Ego-Identity Status:**	187.61	49.07	1	287
• Advanced base	50.89	14.41	1	74
• Postponed base	48.60	13.08	1	72
• Early formed base	44.85	13.04	1	72
• Confused base	43.25	12.68	1	72
**Body management:**	153.12	21.22	78	205
• Appearance evaluation	22.16	4.13	9	34
• Face orientation	42.79	7.45	19	60
• Fitness evaluation	10.05	2.60	2	15
• Fitness orientation	4.15	6.91	26	62
• Mental weight	5.29	2.13	1	10
• Body satisfaction	30.65	8.78	1	45

**Ego-identity status**

Identity is a theory that a person has about himself that is not necessarily fully aware of him, and having it always creates a positive occurrence in the feeling that a person has about himself.

**Advanced base**

People at this site have achieved a solid commitment to various life issues, and have succeeded in succeeding in the search for search and education.

**Postponed base**

The people on this site are lagging behind the commitment and experience of the crisis. They are experiencing periods of crisis and research, but they have not yet reached a specific identity and are actively examining the various cases for reaching a final decision.

**Early formed base**

People at this base have a high commitment, but they do not experience the crisis and exploration. They have accepted the preset identity that the perceived person has set for them, and are more directed by others than themselves.

**Confused base**

These people do not have a commitment or a crisis. They do not have a clear orientation in their lives; they have reached certain values and beliefs, not an attempt to achieve them.

**Body management**

It means monitoring and continuous work and body features

**Appearance evaluation**

Assess various health behaviors such as body building, diets, sports equipment and various body care products.

**Face orientation**

A person׳s perception of her beauty or facial makeup through cosmetic surgeries including nasal surgery, skin rejuvenation, liposuction, lipolysis, abdominal straightening, various injections of beauty.

**Fitness evaluation**

Individual tendency towards fitness and control over the body and attention to the composition, exterior and interior decoration.

**Fitness orientation**

Individual feelings about the appearances of her apparent and sexual beauty.

**Mental weight**

Body monitoring and weight control, and any measures to lose weight or to keep body weight

**Body satisfaction**

The emotion that a person has about his or her body (emotional aspect); each of us has a special feeling about the appearance of our bodies. The emotional image of the body comes from the amount of satisfaction or dissatisfaction with the general appearance, weight, shape, or even certain parts of his body.

**Table 3:** The mean of ego-identity status, body management and its subscales by gender variable.

**Variables**	**Female**	**Male**
	**Mean ± S.D**	**Mean ± S.D**
**Ego-identity status**	43.47 ± 188.52	49.07 ± 187.63
• Advanced base	13.3 ± 51.43	15.75 ± 50.20
• Postponed base	11.85 ± 48.91	14.54 ± 48.19
• Early formed base	11.89 ± 45.10	14.42 ± 44.54
• Confused base	11.20 ± 43.06	14.40 ± 43.50
**Body management:**	20.65 ± 154.75	21.83 ± 151.04
• Appearance evaluation	4.20 ± 22.46	4.03 ± 21.79
• Face orientation	7.33 ± 43.61	7.51 ± 41.75
• Fitness evaluation	2.65 ± 10.07	2.54± 10.03
• Fitness orientation	6.88 ± 41.63	6.91± 42.82
• Mental weight	5.41 ± 2.16	5.13± 2.10
• Body satisfaction	31.56 ± 8.03	29.49 ± 9.57

*Correlation is significant at the 0.05 level (2-tailed).

## 2 Experimental design, materials, and methods

For this data collection, the statistical population consisted of 4200 students at KUMS, of which 360 students were selected from schools of public health, paramedics, nursing, medicine, pharmacy, and dentistry using the Krejcie and Morgan Sampling Formula, and the sampling method was cluster sampling. The data were collected from the 25th May to 30th September 2017. As for data collection, the Ego-Identity Status (EOM-EIS) Questionnaire and Multidimensional Body–Self Relations Questionnaire (MBSRQ) were utilized [1], [2], which were completed by the selected students themselves.

In the Ego-Identity Status (EOM-EIS) Questionnaire, all responses were scored based on Likert scale (1=totally agree to 6=totally disagree), ranging from 16 to 96 points. The interpretation of each scale is as bellows [4]:

• In the confused form, a score of 53 and higher showed the confused identity of individuals;
• In the early formed, a score of 53 and higher showed the early formed identity;
• In the postponed identity, a score of 63 and higher showed the postponed identity in individuals; and
• In the advanced identity, a score of 73 and higher showed the formation of this kind of identity in individuals.
  In addition, the 46-item Multidimensional Body–Self Relations Questionnaire (MBSRQ) was developed by Cash et al. (2015) [2]. In this questionnaire, six aspects were evaluated as follows:
• Appearance Evaluation (7 Questions);
• Appearance Orientation (12 Questions);
• Fitness Evaluation (3 Questions);
• Fitness Orientation (13 Questions);
• Overweight Preoccupation (2 Questions); and
• Body Areas Satisfaction (9 Questions).

All responses were scored based on Likert scale (1=totally agree to 5= totally disagree), ranging from 46 to 230 points. Higher scores on this test were indicative of higher satisfaction with body management.
